# Effect of cilostazol pretreatment on the PARP/AIF-mediated apoptotic pathway in rat cerebral ischemia-reperfusion models

**DOI:** 10.3892/etm.2014.1551

**Published:** 2014-02-17

**Authors:** XIAO-HONG BA, LI-PING CAI, WEI HAN

**Affiliations:** 1 Department of Neurology, The First Affiliated Hospital, Liaoning Medical University, Jinzhou, Liaoning 121001, P.R. China; 2 Department of Neurology, Liaoning Provincial Corps Hospital, Chinese People’s Armed Police Force, Shenyang, Liaoning 110034, P.R. China

**Keywords:** cerebral ischemia-reperfusion, poly ADP-ribose polymerase, apoptosis-inducing factor, cilostazol, apoptosis

## Abstract

The aim of this study was to observe the expression of poly ADP-ribose polymerase (PARP) and apoptosis-inducing factor (AIF) in the CA1 region of the hippocampus and to explore whether cilostazol pretreatment exerts a protective effect on the brain through the PARP/AIF-mediated pathway in a rat model of cerebral ischemia-reperfusion. Rats were randomly divided into three groups: Sham-surgery, ischemia-reperfusion and cilostazol (n=45 rats/group). Rat models of middle cerebral artery occlusion were prepared using a thread occlusion method. Rats in the cilostazol group were administered 30 mg/kg intragastric cilostazol 6 and 2 h before brain ischemia, respectively. Following reperfusion, samples were collected at different time-points (6, 24 and 72 h) and each group was further subdivided into three subgroups (n=15 rats/subgroup). Apoptosis was measured using the terminal deoxynucleotidyl-transferase-mediated dUTP nick end labeling method. The protein expression levels of AIF and PARP were detected using western blot analysis and the expression levels of AIF mRNA were determined using the reverse transcription-polymerase chain reaction. AIF nuclear translocation occurred following local cerebral ischemia-reperfusion injury. Apoptosis, levels of AIF and PARP protein expression and levels of AIF mRNA expression were significantly increased in the ischemia-reperfusion group compared with the sham-surgery group (P<0.05). However, apoptosis and the expression levels of AIF protein, PARP protein and AIF mRNA at different time-points were significantly decreased in the cilostazol group compared with the ischemia-reperfusion group (P<0.05). In conclusion, cilostazol has a protective effect on rat cerebral ischemia-reperfusion injury, and acts by inhibiting nerve cell apoptosis by preventing the excessive activation of PARP and AIF nuclear translocation.

## Introduction

Cilostazol, a selective inhibitor of phosphodiesterase III, is able to inhibit PDE-3 activity with the increase in intracellular cAMP concentration which regulates cell function, inhibits platelet aggregation, prevents thrombosis, relaxes vascular smooth muscle, inhibits cell proliferation and regulates blood lipid level. Apoptosis-inducing factor (AIF) is associated with nerve cell death. The excessive activation of poly ADP-ribose polymerase (PARP) sends the nuclear signal to the mitochondrion, triggering AIF release from the mitochondrion to the cell nucleus with chromatin condensation and DNA fragments. Cilostazol has protective effects on cerebral ischemia-reperfusion injury and the protective effects are associated with PARP inhibition (1).

The main apoptosis-mediated signaling pathways include the caspase-8-mediated death receptor pathway, the caspase-9-mediated mitochondrial pathway and the caspase-12-mediated endoplasmic reticulum stress pathway. However, non-caspase-mediated apoptotic pathways also exist, including the poly ADP-ribose polymerase (PARP)/apoptosis-inducing factor (AIF)-mediated apoptotic pathway (2–4). The PARP/AIF-mediated apoptotic pathway is an important pathway that is present in numerous eukaryotic organisms. In this study, the effects of cilostazol on the PARP/AIF-mediated apoptotic pathway in a rat model of cerebral ischemia-reperfusion injury were investigated, providing an experimental basis for the prevention and treatment of cerebrovascular disease.

## Materials and methods

### Ethical approval

All study methods were approved by the Ethics Committee of the First Affiliated Hospital, Liaoning Medical University (Jinzhou, China).

### Animal and reagents

Sprague Dawley rats (n=135), weighing between 280 and 320 g, were purchased from the Experimental Animal Center, Liaoning Medical University. Cilostazol was purchased from Otsuka Pharmaceutical Company (Suzhou, China). Rabbit anti-mouse AIF polyclonal antibody was purchased from Bioss Company (Beijing, China). Antibodies against Histone H1 was purchased from Santa Cruz (Dallas, TX, USA) and antibodies against PARP was purchased from Alexis (New York, NY, USA). Mouse anti-apoptotic reagent was purchased from Alexis Biochemicals (Ann Arbor, MI, USA). The immunohistochemical staining kit was purchased from Wuhan Boster Biological Engineering Co., Ltd (Wuhan, China). The AIF and β-actin primers were synthesized by Shanghai Generay Biotechnology Co., Ltd (Shanghai, China), and the total RNA extraction kit was purchased from Shanghai Generay Biotechnology Co., Ltd.

### Grouping and model preparation

The rats were randomly divided into three groups: Sham-surgery, ischemia-reperfusion and cilostazol (n=45/group). Rat models of right middle cerebral artery occlusion were prepared using the thread occlusion method (5). The blood flow was restored 2 h after ischemia. Rats that exhibited Horner’s sign, adduction and flexion of the left forelimb, and that had a tendency to fall to the left or crawl in a counterclockwise direction. For the sham-surgery group, the thread was inserted into the right middle cerebral artery and then immediately removed. For the cilostazol group, 30 mg/kg intragastric cilostazol was administered 6 and 2 h before brain ischemia, respectively. Samples were collected at different time-points (6, 24 and 72 h) after reperfusion, and each group was further divided into three subgroups (n=15/subgroup).

### Brain samples

At the different time-points after reperfusion (6, 24 and 72 h), five rats were taken from each subgroup. The brain was removed from each rat following decapitation and divided into four equal parts (A, B, C and D) from the frontal region to the occipital region. Part C was dehydrated with alcohol, embedded in paraffin and sectioned at a thickness of 5 μm for immunohistochemical staining. A further 10 rats from each subgroup were sacrificed at different time-points (6, 24 and 72 h) after reperfusion, and the hippocampus of each rat was placed in liquid nitrogen. The hippocampi of five of the rats were used for western blot analysis, whilst the hippocampi of the remaining five rats were used for reverse transcription-polymerase chain reaction (RT-PCR).

### Analysis of nerve cell apoptosis using the terminal deoxynucleotidyl-transferase-mediated dUTP nick end labeling (TUNEL) method

Nerve cell apoptosis was detected using a TUNEL kit provided by Sigma (St. Louis, MO, USA). One CA1 hippocampus slice was randomly selected from each rat, and five fields were then randomly selected from the CA1 hippocampus slice (Olympus, Hatagaya, Japan). The number of TUNEL-positive cells was counted in the five fields. Since there were five rats in each subgroup, the average number of TUNEL-positive cells in 25 fields was calculated.

### Western blot analysis

Cell lysis solution was added to the samples and nuclei were extracted from the cells using the differential centrifugation method. Nuclear lysates were added prior to centrifugation in order to extract nucleoprotein. The quantity of protein extracted was determined using the Bradford assay. Western blotting was performed according to a previously described method (6). The electrophoresis pattern was analyzed using a gel imaging analysis system (GDS-8000 type; UVP, Upland, CA, USA). The absorbance of AIF and PARP was detected and histone H1 was used as an internal control. AIF and PARP protein expression was calculated relative to the control, histone H1.

### Analysis of AIF mRNA expression using RT-PCR

Hippocampal tissue was stored at −80°C for future use. Total RNA was extracted using the Trizol method. Total RNA underwent reverse transcription for the preparation of cDNA templates. PCR amplification was performed using 2 μl cDNA, and β-actin was used as an internal control. AIF primer sequences were as follows: Forward, 5′-CCCCGATGTTGGCTATGA-3′ and reverse, 5′-TCCTGACTGCTCTGTGGC-3′, with an amplified fragment of 115 base pairs (bp). β-actin primer sequences were as follows: Forward, 5′-CACCAACTGGGACGACAT-3′ and reverse, 5′-ACAGCCTGGATAGCAACG-3′, with an amplified fragment of 189 bp. The PCR conditions were as follows: Pre-denaturing at 94°C for 5 min, denaturing at 94°C for 30 sec, reannealing at 54°C for 30 sec and elongation at 72°C for 5 min. PCR products were then analyzed using 30 g/l agarose gel electrophoresis. Image scanning was performed using Gene Tools software (Syngene, Frederick, MD, USA). The level of AIF mRNA was calculated according to the gray-level value.

### Statistical analysis

Statistical analysis was performed using SPSS 13.0 software (SPPS, Inc., Chicago, IL, USA). Data are expressed as the mean ± standard deviation. A Student’s t-test was used for the comparison between two samples and repeated measures analysis of variance was used in the comparison of multiple samples. P<0.05 was considered to indicate a statistically significant difference.

## Results

### Effect of cilostazol pretreatment on nerve cell apoptosis

In the sham-surgery group, only a few apoptotic cells were observed at the different time-points and no statistical difference was found in the number of apoptotic cells among the different time-points (P>0.05). In the ischemia-reperfusion group, the number of apoptotic cells was markedly increased 24 h after reperfusion and was decreased 72 h after reperfusion compared with the 6-h time-point. However, compared with the sham-surgery group, the apoptosis rate in the ischemia-reperfusion group was significantly increased at all time-points (P<0.05). Compared with the ischemia-reperfusion group, the apoptosis rate was significantly decreased in the cilostazol group at all time-points (P<0.05). For the ischemia-reperfusion and cilostazol groups, the apoptosis rate was highest after 24 h (P<0.05) (Table I).

**Figure 1:**
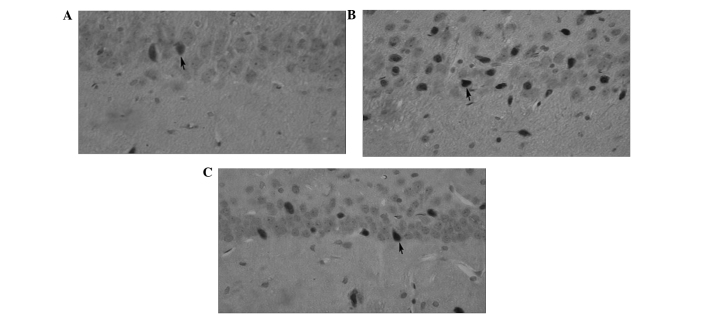
Terminal deoxynucleotidyl-transferase-mediated dUTP nick end labeling-positive cells in the hippocampus at 24 h after reperfusion in each group (magnification ×200). The black arrow indicates apoptotic cells. (A) Sham-surgery group; (B) ischemia-reperfusion group; (C) cilostazol group.

At the 24-h time-point, a few TUNEL-positive cells were observed in the sham-surgery group. The number of TUNEL-positive cells observed in the ischemia-reperfusion group was markedly increased in comparison with the sham-surgery group and the number of TUNEL-positive cells observed in the cilostazol group was markedly decreased compared with the ischemia-reperfusion group (Fig. 1).

### Effects of cilostazol pretreatment on AIF nuclear translocation and PARP

**Figure 2:**
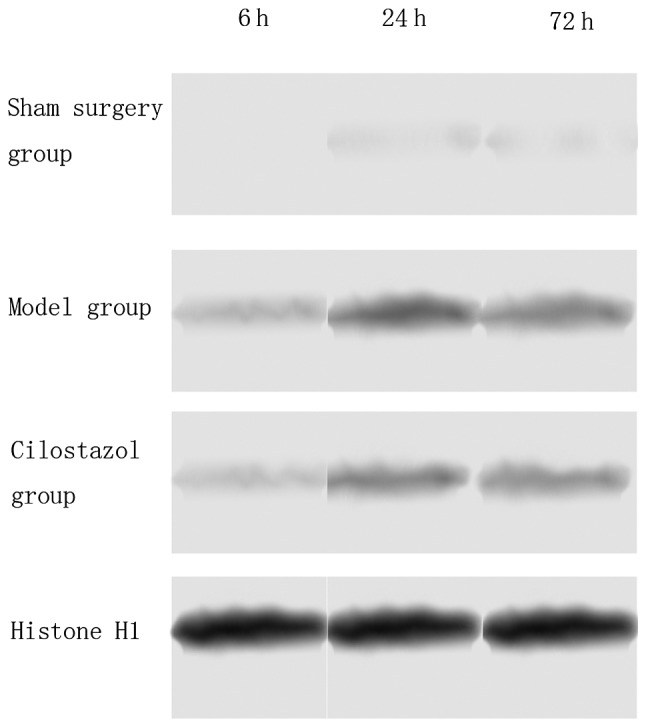
Apoptosis-inducing factor protein expression in the CA1 region of the hippocampus at different-time points in each group.

**Figure 3:**
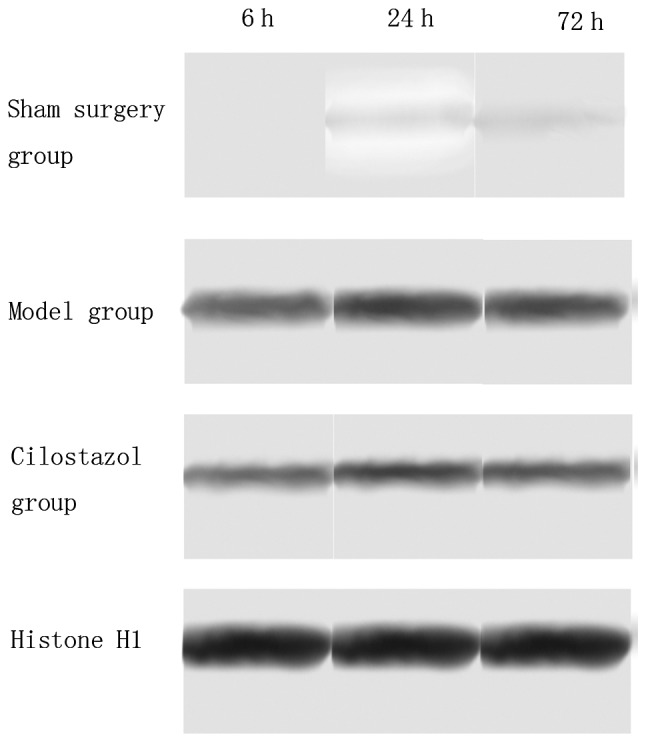
Poly ADP-ribose polymerase protein expression in the CA1 region of the hippocampus at different time-points in each group.

The results of the western blot analysis indicated that, in the sham-surgery group, there was no PARP and AIF nuclear translocation; however, in the ischemia-reperfusion group, PARP and AIF nuclear translocation occurred 6 h after reperfusion, was markedly increased 24 h after reperfusion and then markedly decreased after 72 h. Compared with the ischemia-reperfusion group, PARP and AIF nuclear translocation was significantly decreased in the cilostazol group at all time-points (P<0.05). In the ischemia-reperfusion and cilostazol groups, PARP and AIF nuclear translocation at 24 h was the highest among the different time-points (P<0.05) (Tables II and III, Figs. 2 and 3).

### Effect of cilostazol pretreatment on AIF mRNA expression levels

**Figure 4:**
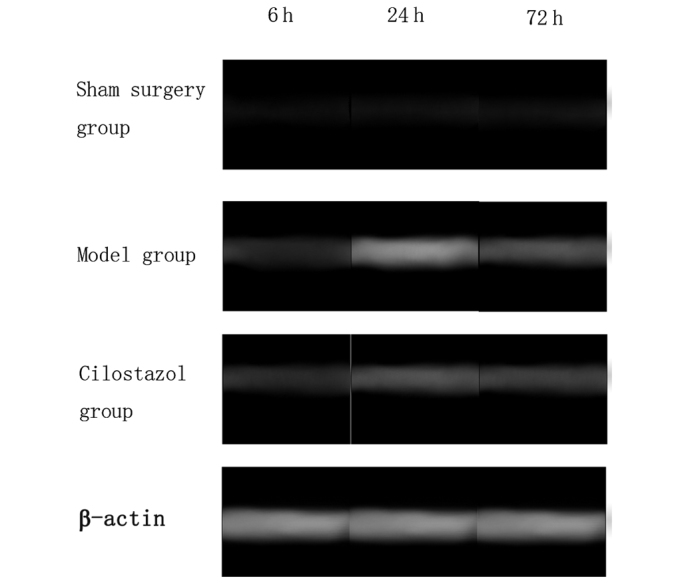
Apoptosis-inducing factor mRNA expression in the CA1 region of the hippocampus at different time-points in each group.

In the sham-surgery group, there was little expression of AIF mRNA at all time-points. In the ischemia-reperfusion group, AIF mRNA expression was observed 6 h after reperfusion, and was markedly increased 24 h after reperfusion, prior to being reduced again 72 h after reperfusion. However, compared with the sham-surgery group, a significant difference was observed in AIF mRNA expression in the ischemia-reperfusion group at all time-points (P<0.05). Compared with the ischemia-reperfusion group, AIF mRNA expression was significantly decreased in the cilostazol group at all time-points (P<0.05). In the ischemia-reperfusion and cilostazol groups, AIF mRNA expression was highest 24 h after reperfusion (P<0.05) (Table IV and Fig. 4).

## Discussion

Aspirin is an effective and economical antiplatelet drug that is widely used in clinical practice. However, certain patients have aspirin resistance or are not able to tolerate the drug. Cilostazol is a novel antiplatelet drug that exerts similar therapeutic effects, but has a reduced risk of bleeding and cerebral infarction compared with aspirin (7). Cilostazol is a selective inhibitor of the cyclic nucleotide PDE-3 and increases cyclic adenosine monophosphate (cAMP) levels in platelets and vascular endothelial cells by inhibiting PDE-3 activity and cAMP degradation. This inhibits platelet aggregation, relaxes the vasculature and regulates blood lipids, preventing atherosclerosis and vascular occlusion (8). Cilostazol has generated significant interest in the treatment of cerebrovascular disease. It has been proposed that the protective effect of cilostazol on cerebral ischemia-reperfusion injury is associated with the inhibition of apoptosis (1).

The present study demonstrated that, in the ischemia-reperfusion group, the number of apoptotic cells was increased 24 h after reperfusion compared with the 6-h time-point. A significant difference was observed in the apoptosis rate in the ischemia-reperfusion group at all time-points compared with the sham-surgery group (P<0.05). However, the apoptosis rate was significantly decreased in the cilostazol group at all time-points compared with the ischemia-reperfusion group (P<0.05). In the ischemia-reperfusion and cilostazol groups, the apoptosis rate at 24 h was the highest among the different time-points (P<0.05). The results demonstrated that cilostazol was able to inhibit apoptosis caused by cerebral ischemia-reperfusion injury; however, the mechanism underlying the action of cilostazol was not clear. In order to investigate this further, the PARP/AIF apoptotic pathway was studied. PARP, a type of ribozyme, identifies and repairs DNA single-strand breaks in order to maintain genome integrity, and has beneficial and harmful effects on cell injury and repair. In mild ischemia, nerve cells survive as a result of PARP repairing damaged DNA; however, in severe ischemia, there is significant DNA damage, which causes the excessive activation of PARP, leading to the consumption of nicotinamide adenine dinucleotide (NAD), the formation of NAD^+^ and the consumption of ATP (9). Energy depletion leads to cell damage, apoptosis or necrosis (10). In this study, the effects of cilostazol on PARP expression were observed 6, 24 and 72 h after cerebral ischemia-reperfusion. The results indicated that cerebral ischemia-reperfusion injury activates PARP expression, since PARP expression was markedly increased 24 h after cerebral ischemia-reperfusion injury, and that cilostazol is capable of decreasing PARP expression. The results suggest that the anti-apoptotic effect of cilostazol is associated with the downregulation of PARP expression in cerebral ischemia-reperfusion injury.

PARP is capable of inducing changes in mitochondrial membrane permeability, leading to the release of AIF. The excessive activation of PARP located in the mitochondria directly causes mitochondrial injury and the release of pro-apoptotic proteins, leading to AIF nuclear translocation (1). Apoptotic signals cause the excessive activation of PARP which results in AIF release from the mitochondrion to the cell nucleus (11). The excessive activation of PARP consumes NAD^+^, leading to energy depletion (12). In the present study, the effect of cilostazol on AIF protein expression in cerebral ischemia-reperfusion was observed. The results showed that AIF expression in the ischemia-reperfusion group was increased 24 h after reperfusion compared with that at the 6-h time-point, prior to decreasing again 72 h after reperfusion. This may be due to the fact that AIF in the mitochondria has a protective role in early reperfusion; with the prolongation of reperfusion, AIF was released from the mitochondria and mitochondrial integrity was damaged. This result suggests that, in order to reduce AIF nuclear translocation, drug intervention should be adopted within 24 h after reperfusion. Compared with the ischemia-reperfusion group, AIF expression was significantly decreased in the cilostazol group, demonstrating that cilostazol was able to inhibit AIF nuclear translocation. The results also indicated that AIF mRNA expression was reduced in the sham-surgery group compared with the ischemia-reperfusion and cilostazol groups. In the ischemia-reperfusion group, AIF mRNA expression was observed 6 h after reperfusion, reaching a peak 24 h after reperfusion and then decreasing again after 72 h. This suggests that apoptotic signals stimulate AIF transcription. The dynamic changes in AIF mRNA expression were consistent with those in nerve cell apoptosis, suggesting that AIF gene transcription is involved in apoptosis. Compared with the ischemia-reperfusion group, AIF mRNA expression was significantly decreased in the cilostazol group at all time-points, suggesting that cilostazol is capable of inhibiting AIF expression at the transcriptional level, and then inhibiting apoptosis to exert protective effects on the brain.

In conclusion, in a rat model of cerebral ischemia-reperfusion, apoptotic cells, increased expression of PARP, NAD depletion and AIF nuclear translocation were observed. Cilostazol was found to inhibit apoptosis, the excessive activity of PARP, NAD depletion and AIF nuclear translocation, suggesting that the anti-apoptotic effects of cilostazol may be associated with the inhibition of excessive PARP activation and AIF mRNA expression, as well as AIF nuclear translocation.

## Figures and Tables

**Table I:** TUNEL-positive cells in the CA1 region of the hippocampus at different time-points in each group (n=5).

	TUNEL-positive cells (%)				
			
Groups	6 h	24 h	72 h	F-value	P-value
Sham-surgery	0.949±0.162	1.031±0.153	1.146±0.494	6.624	0.076
Ischemia-reperfusion	23.845±3.633^b^	48.531±7.810^b^,^c^	15.029±3.718^b^	3.626	0.044
Cilostazol	12.763±3.470^a^	25.678±9.206^a^,^c^	4.341±2.929^a^	12.413	<0.001
F-value	7.819	13.448	3.666	-	-
P-value	0.003	<0.001	0.043	-	-

a P<0.05 compared with the ischemia-reperfusion group;

b P<0.05 compared with the sham-surgery group;

c P<0.05 compared with values at different time-points within the ischemia-reperfusion and the cilostazol groups.

TUNEL-positive cell data are presented as the mean ± standard deviation. TUNEL, terminal deoxynucleotidyl-transferase-mediated dUTP nick end labeling.

**Table II:** AIF protein expression in the CA1 region of the hippocampus at different time-points in each group (n=5).

	AIF protein expression (%)				
			
Groups	6 h	24 h	72 h	F-value	P-value
Ischemia-reperfusion	0.721±0.005	1.240±0.041^b^	0.621±0.015	14.355	<0.001
Cilostazol	0.715±0.008^a^	0.869±0.016^a^,^b^	0.411±0.007^a^	28.561	<0.001
T-value	3.785	5.148	3.640	-	-
P-value	0.007	0.010	0.008	-	-

a P<0.05 compared with the ischemia-reperfusion group;

b P<0.05 compared with values at different time-points within the ischemia-reperfusion and the cilostazol groups.

Protein expression data are presented as the mean ± standard deviation. AIF, apoptosis-inducing factor.

**Table III:** PARP protein expression in the CA1 region of the hippocampus at different time-points in each group (n=5).

	PARP protein expression (%)				
			
Groups	6 h	24 h	72 h	F-value	P-value
Ischemia-reperfusion	53.001±6.477	79.251±7.073^b^	33.001±8.521	78.451	<0.001
Cilostazol	49.243±5.025^a^	61.743±15.54^a^,^b^	21.743±4.696^a^	47.263	<0.001
T-value	10.503	2.487	3.841	-	-
P-value	<0.001	0.042	0.006	-	-

a P<0.05 compared with the ischemia-reperfusion group;

b P<0.05 compared with values at different time-points within the ischemia-reperfusion and the cilostazol groups.

Protein expression data are presented as the mean ± standard deviation. PARP, poly ADP-ribose polymerase.

**Table IV:** mRNA expression of AIF in the CA1 region of the hippocampus at different time-points in each group (n=5).

	AIF mRNA expression (%)				
			
Groups	6 h	24 h	72 h	F-value	P-value
Sham-surgery	0.175±0.031	0.220±0.106	0.155±0.034	16.928	0.052
Ischemia-reperfusion	0.955±0.084^b^	2.056±0.367^b^,^c^	0.966±0.093^b^	9.345	0.001
Cilostazol	0.575±0.330^a^,^b^	1.378±0.171^a^,^b^,^c^	0.596±0.389^a^,^b^	5.809	0.010
F-value	5.342	5.470	4.369	-	-
P-value	0.013	0.012	0.026	-	-

a P<0.05 compared with the ischemia-reperfusion group;

b P<0.05 compared with the sham-surgery group;

c P<0.05 compared with values at different time-points within the ischemia-reperfusion and the cilostazol groups.

Data are presented as the mean ± standard deviation. AIF, apoptosis-inducing factor.
